# Cases of Asthma Successfully Treated by Nitric Acid

**Published:** 1850-11

**Authors:** T. S. Hopkins

**Affiliations:** Bethel, Glynn county, Georgia


### Part 3.—Selections.

### ARTICLE I.

*Cases of Asthma successfully treated by Nitric Acid.*—By T. S. Hopkins, M. D., of Bethel, Glynn county, Georgia.

The following five cases of Asthma, cured by the use of Nitric Acid, have occurred in my practice since the summer of 1847. It is not my desire to attempt any explanation as to the modus operandi of this medicine in the above disease. Its beneficial effects were accidentally discovered, and, after a fair trial in five consecutive cases, with the most entire success, I am induced to bring it to the notice of the profession, trusting that in other hands than my own it may prove a potent agent for the relief of a disease which so often resists the best directed treatment. Several of these cases were from twenty-five to thirty-five miles distant from my office. Most of them were not seen by me from the time of my first visit and prescription until a cure had been effected. I describe them as I found them at the time of my visit, and from the history given by parents and masters, which I think can be depended upon.

Case I.—Emma, negro girl, aged five years, belonging to Mr. T. G., had been asthmatic *almost* from birth. Nightly paroxysms of dyspnœa, cough, &c., were represented as most distressing. During the day she would be up and about the yard with the other children. At the time that I saw her, her respiration was somewhat embarrassed, with slight elevation of the shoulders during inspiration, and a very distinct mucous râle. Her appetite was impaired and her countenance cheerless.

I ordered nitric acid, three drops, to be increased to five, three times daily, in a wineglassful of sugared water. A month elapsed before I again saw this patient, at which time every symptom of disease had disappeared. I prescribed for her in December, 1847, and up to date no symptom of asthma has returned.

Case II.—I was called in November, 1848, to see W. S., aged about six years, son of a planter of Glynn county. I was informed that this boy had been a subject of asthma for four or five years; that no expense had been spared in seeking for relief in his case; and that all the efforts of the best physicians in the neighborhood in which he had resided, had been in vain. When I saw him, he had cough, slight dyspnœa, with mucous râle distinctly audible at the distance of several feet. By walking up and down the steps of the house once or twice, the difficulty of breathing and cough would be much increased. He was very lively and cheerful, with a good appetite, and had had no fever for months. His father informed me that, upon the least *exposure* during the day, he would be attacked at night with the most fearful symptoms. Wheezing, panting, incessant cough, distressing dyspnœa, with impending suffocation, were the inevitable consequences (at night) of exposure during the day.

I ordered nitric acid, five drops, three times daily, in a wineglass of sugared water, with a strict avoidance of exposure.

In a fortnight, he had been so much relieved that his parents imagined him cured, and discontinued the acid. In a few days he relapsed, and I was again sent for. I ordered the acid to be continued, as before. and in one month from the time of my first visit he was *cured.*

Up to date there has been no return of disease.

This boy's father died of phthisis pulmonalis. Several of his mother's family have died of the same disease; and all of *his* brothers and sisters, without an exception, have in early childhood been sufferers from enlargement of tonsils and ulcerated sore throat. He is now the picture of health.

Case III.—A mulatto girl, belonging to Mr. W. D. T., aged four years. This was a case of congenital asthma.

The symptoms in this and the following case (aged about four years) were so similar to those of Case I. that I shall not describe them.

The treatment was nitric acid, three drops three times daily, in a wineglass of sugared water.

This case resisted the treatment rather longer than the other cases, but was *cured* in about six weeks.

In Case IV., the immediate effects of the acid were perceived in a few days; and in eight or ten days she was cured.

Case V.—I never saw. It was a negro girl belonging to Mr. C., of Camden county. She was seven years of age.

Her master applied to me for a prescription, after giving some of the most prominent symptoms, which convinced me that the case was asthma, as he had declared it to be.

I prescribed five drops of nitric acid, three times daily.

I heard nothing more of this case for several months after I had prescribed, when I was informed by her master that she was well.

A sufficient time has elapsed, in all these cases, to convince me that they have been radically cured.

Should you deem the above remarks and cases worthy a place in your valuable journal, you will please to insert them. It is at least *new* in the treatment of asthma.—*Amer. Jour. Med. Sci.*

